# Conditional HIF-1α Expression Produces a Reversible Cardiomyopathy

**DOI:** 10.1371/journal.pone.0011693

**Published:** 2010-07-21

**Authors:** Raffi Bekeredjian, Chad B. Walton, Keith A. MacCannell, Jennifer Ecker, Fred Kruse, Joel T. Outten, David Sutcliffe, Robert D. Gerard, Richard K. Bruick, Ralph V. Shohet

**Affiliations:** 1 Department of Medicine, University of Hawaii, Honolulu, Hawaii, United States of America; 2 Department of Cardiology, University of Heidelberg, Heidelberg, Germany; 3 Department of Biochemistry, University of Texas Southwestern Medical Center, Dallas, Texas, United States of America; 4 Department of Medicine, University of Texas Southwestern Medical Center, Dallas, Texas, United States of America; Case Western Reserve University, United States of America

## Abstract

**Background:**

The response to hypoxia in tissues is regulated by the heterodimeric transcription factor Hypoxia Inducible Factor-1 (HIF-1).

**Methodology/Principal Findings:**

We have created a strain of mice with inducible cardiomyocyte-specific expression of a mutated, oxygen-stable, form of HIF-1α. Cardiac function steadily decreased with transgene expression, but recovered after the transgene was turned off. Using long-oligo microarrays, we identified 162 transcripts more than 3-fold dysregulated in these hearts after transgene expression. Among the down-regulated genes the transcript for SERCA was reduced 46% and the protein 92%. This led us to an evaluation of calcium flux that showed diminished reuptake of cytoplasmic calcium in myocytes from these hearts, suggesting a mechanism for cardiac dysfunction.

**Conclusions/Significance:**

These results provide a deeper understanding of transcriptional activity of HIF in the heart, and show that enhanced HIF-1 activity is sufficient to cause contractile dysfunction in the adult heart. HIF is stabilized in the myocardium of patients with ischemic cardiomyopathy, and our results suggest that HIF could be contributing directly to the contractile dysfunction in this disease.

## Introduction

HIF-1 is a heterodimeric transcription factor that regulates a wide range of angiogenic, metabolic, and oxygen transport related genes [1]. While the HIF-1β subunit, also known as the aryl hydrocarbon receptor nuclear translocator, (ARNT) is a constitutively expressed protein, HIF-1α is rapidly degraded in normoxic conditions [2]. In hypoxic conditions, HIF-1α is stable and can bind to HIF-1β [2] to form a dimer that binds to the hypoxia response element (HRE) in HIF-1 regulated genes [3]. The destabilization of HIF-1α by oxygen is understood in detail. In normoxic conditions, two proline residues (Pro402 and Pro564) [4], are hydroxylated by specific prolyl hydroxylases [5]–[8]. These hydroxylated sites are recognized by the von-Hippel Lindau tumor suppressor (pVHL)[8], which ultimately leads to proteosomal degradation [5], [9]–[15]. Another oxygen dependent control acting to limit the activity of HIF-1α involves hydroxylation of an asparagine residue (Asn803) by Factor Inhibiting HIF (FIH) [16]–[18]. This prevents the HIF-1α C-terminal activation domain from interacting with the transcriptional co-activating protein p300 [16], in turn limiting the transcriptional activity of HIF-1 in a normoxic environment [19].

To study the action of HIF-1 in a normoxic environment, it would be useful to disable this oxygen-related regulation. Substitution of Pro564 and Pro402 abrogates HIF-1α/pVHL interaction [8] and the subsequent ubiquitinylation and degradation of HIF-1α [6], [20]. It has also been shown that enhanced transcriptional activity, due to association of HIF-1α to p300, is obtained in a normoxic environment by double substitution of Pro564 and Asn803 with alanine [16].

For our studies we have created a transgenic mouse line to directly test the effect of HIF-1α in the hearts of adult animals in a normoxic environment. A transgene containing the human HIF-1α cDNA with alanine substitutions at Pro402, Pro564, and Asn803 (denoted HIF-1α-PPN), was generated. Since VEGF is upregulated by HIF-1α [21] and VEGF overexpression during fetal [22] and postnatal [23] stages of development is lethal, we used a tetracycline inducible construct (tet-off system) to obtain tight exogenous regulation of transgene expression. This also allows us to investigate the effects of enhancing HIF-1 activity in the adult animal, avoiding any developmental effects, and modeling the potential use of HIF-1α for therapy of ischemia. We then evaluated the resulting cardiac phenotype and explored the underlying biochemical, physiological, and transcriptional results of HIF-1 stabilization.

Recently, others have shown that a knock-out of the gene for the Von Hippel Lindau protein, which should also lead to stabilization of HIF, also causes depressed ventricular function [24]. The ventricular dysfunction is ameliorated in a strain with a knock-out of HIF-1α, further implicating HIF in the dysfunction. This absence of VHL, occurring throughout the life of the animal, causes damage to the myocardium that leads to a dilated cardiomyopathy with a variety of marked histological findings including lipid accumulation, myocyte loss, fibrosis, and even malignant transformation. We do not see similar changes with brief expression of HIF in the adult, but do find remarkable angiogenesis and ventricular dysfunction, which is readily reversible with cessation of transgene expression. Deletion of Prolyl Hydroxylase 2 (PHD2), the enzyme thought to be most important for HIF-1α degradation, also leads to reduced contractility [25] suggesting that stabilization of HIF may impair cardiac function, although other effects of prolyl hydroxylation could be contributing to this effect.

Other studies suggest that exogenous HIF-1 can mediate potentially beneficial effects on vascular growth [26]. In a conventional, unregulated, transgenic model, HIF-1α expression has recently been shown to attenuate cardiac dysfunction following myocardial infarction [27] and it seems reasonable that a transcription factor that responds to decreased oxygen delivery would ameliorate the repercussions of hypoxia by improving blood delivery and modulating oxidative metabolism. Nonetheless, several lines of evidence point to an early decrement in ventricular function mediated by HIF. Interestingly, the condition of ischemic cardiomyopathy would be expected to stabilize HIF [28], which could thereby contribute to the ventricular dysfunction of this common cardiac disorder. It is a clinical observation that the degree of ventricular dysfunction in ischemic cardiomyopathy is often not closely related to the severity of epicardial coronary disease. Perhaps this discordance is a function of the stabilization of HIF, which would relate to the degree of hypoxia in living cardiac myocytes, rather than the amount of damage wrought by previous infarction. The reversible nature of the defect after HIF induction suggests the potential contractile benefit that might accrue from strategies directed towards relief of ischemia. Our model has also allowed us to identify altered calcium handling as one of the principal defects contributing to ventricular dysfunction in this model, and a potential target for therapeutic manipulation.

## Results

### Biological Activity of HIF-1α-PPN

The transcriptional activity of the mutated HIF-1α was assessed by co-transfection of the HIF-1α-PPN expression plasmid with an HRE-luciferase reporter construct into HeLa cells. Co-transfection of a plasmid containing wild-type HIF-1α caused negligible induction of the HRE-luciferase reporter system under normoxic conditions. However, increasing amounts of HIF-1α-PPN under normoxic conditions induced luciferase activity to levels comparable to those obtained from the wild-type protein under hypoxic conditions (Table 1).

**Table 1:** Luciferase activity for increasing WT and HIF-1α-PPN transfection compared to that expressed in normoxic and hypoxic conditions.

	Induction			
DNA [ng]	WT HIF-1α transfection	HIF-1α-PPN transfection	normoxia	hypoxia
			1	104.9
1	0.1	3.7		
3	1	17.8		
10	1	30.7		
30	3	87.5		
100	0.1	58.8		

Each number represents the average of three independent measurements, normalized to normoxia. WT: wildtype.

**Figure 1:**
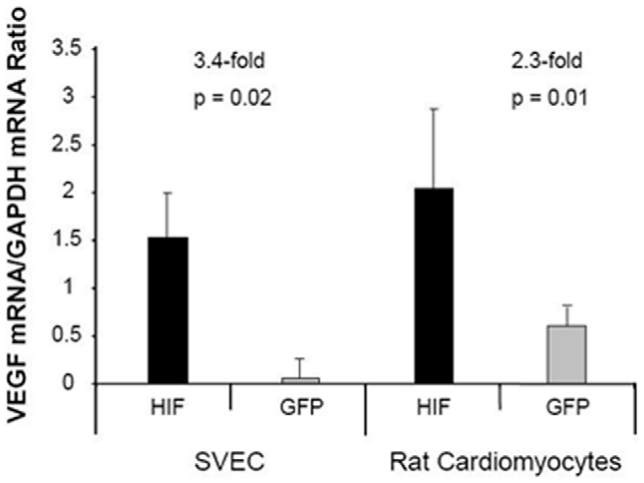
VEGF expression in SVEC and cardiomyocyte cell culture 72 hrs after infection with adenoviruses encoding HIF-1αPPN or GFP.

HIF-1α-PPN was also tested for its ability to elicit authentic HIF-1α transcriptional regulation in our anticipated target cell types, endothelial cells and cardiac myocytes. SV-40 transformed mouse vascular endothelial cells (SVEC) and neonatal rat cardiac myocytes were infected with either AdCMV-HIF-1α-PPN or AdCMV-GFP. HIF-1α-PPN was robustly expressed in the SVEC cells under normoxic conditions (not shown). The transcript for VEGF-A, a known target gene of HIF-1α, was measured by real-time PCR. HIF-1α-PPN expression produced increased abundance of the endogenous VEGF-A transcript in both SVEC cells and cardiac myocytes (Figure 1).

### Conditional Expression of HIF-1α-PPN in Transgenic Mice

**Figure 2:**
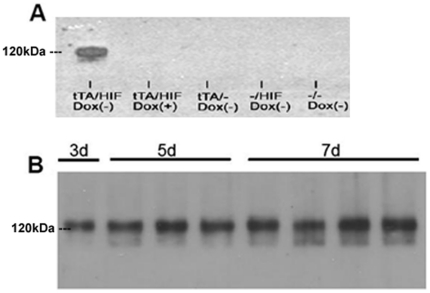
Western blot of anti-HA immunoprecipitated HIF-1α-PPN from mouse hearts induced by doxycycline withdrawal for (A) 14 days and (B) 3, 5, and 7 days. Each lane contains cardiac lysate from a different animal.

Initially, HIF-1α transgene expression in the hearts of tTA/HIF-1α-PPN mice was verified by removing doxycycline from drinking water for 14 days. Immunoprecipitation of HA tagged HIF-1α-PPN from heart protein lysates demonstrated expression of the transgene (Figure 2A). Importantly, tTA/HIF-1α-PPN mice maintained on doxycycline showed no band on the Western blot, indicating tight control of HIF-1α-PPN expression. Doxycycline was then withdrawn from tTA/HIF-1α-PPN mice for 3, 5, and 7 days to test when the HIF-1α-PPN transgene was maximally expressed. Western blotting of immunoprecipitated HIF-1α-PPN demonstrated that the abundance of transgenic protein is essentially maximal by 3 days (Figure 2B). Similarly, real-time PCR determined that HIF-1α-PPN mRNA levels reached a peak by 3 days after doxycycline omission, with no further increase after 7 days of doxycycline withdrawal (data not shown).

### Ventricular function of tTA/HIF-1α-PPN Mice

**Figure 3:**
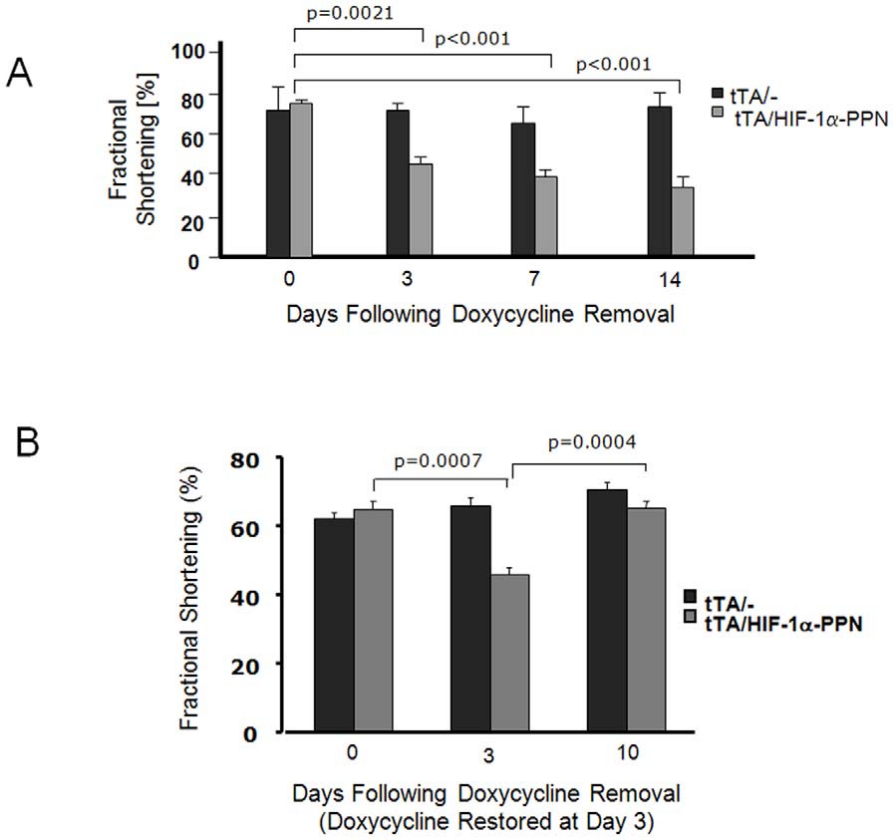
Echocardiographic fractional shortening of tTA/HIF-1α-PPN vs. tTA mouse hearts at (A) 0, 3, 7, and 14 days after doxycycline removal and (B) after 3 days of transgene induction followed by 7 days of recovery.

Echocardiographic analysis of tTA/HIF-1α-PPN mice 14 days after doxycycline withdrawal showed marked dilation of the left ventricle (LVEDD: 3.05±0.4 mm vs. 2.09±0.06 mm; p = 0.007), a substantial decline in fractional shortening (36±5% vs. 73±5%; p = 0.0004; Figure 3A), but only a modest and statistically non-significant increase in posterior wall thickness (0.88±0.1 mm vs. 0.97±0.1 mm; p = 0.21). Even after only 3 days of HIF-1α-PPN expression, ventricular function was impaired in tTA/HIF-1α-PPN vs. tTA mice (fractional shortening decreased to 44±5% vs. 65±5%; p = 0.0004). Importantly, after doxycycline was restored to the drinking water for 7 days ventricular function returned to normal (Figure 3B).

### Morphological analyses of hearts

**Figure 4:**
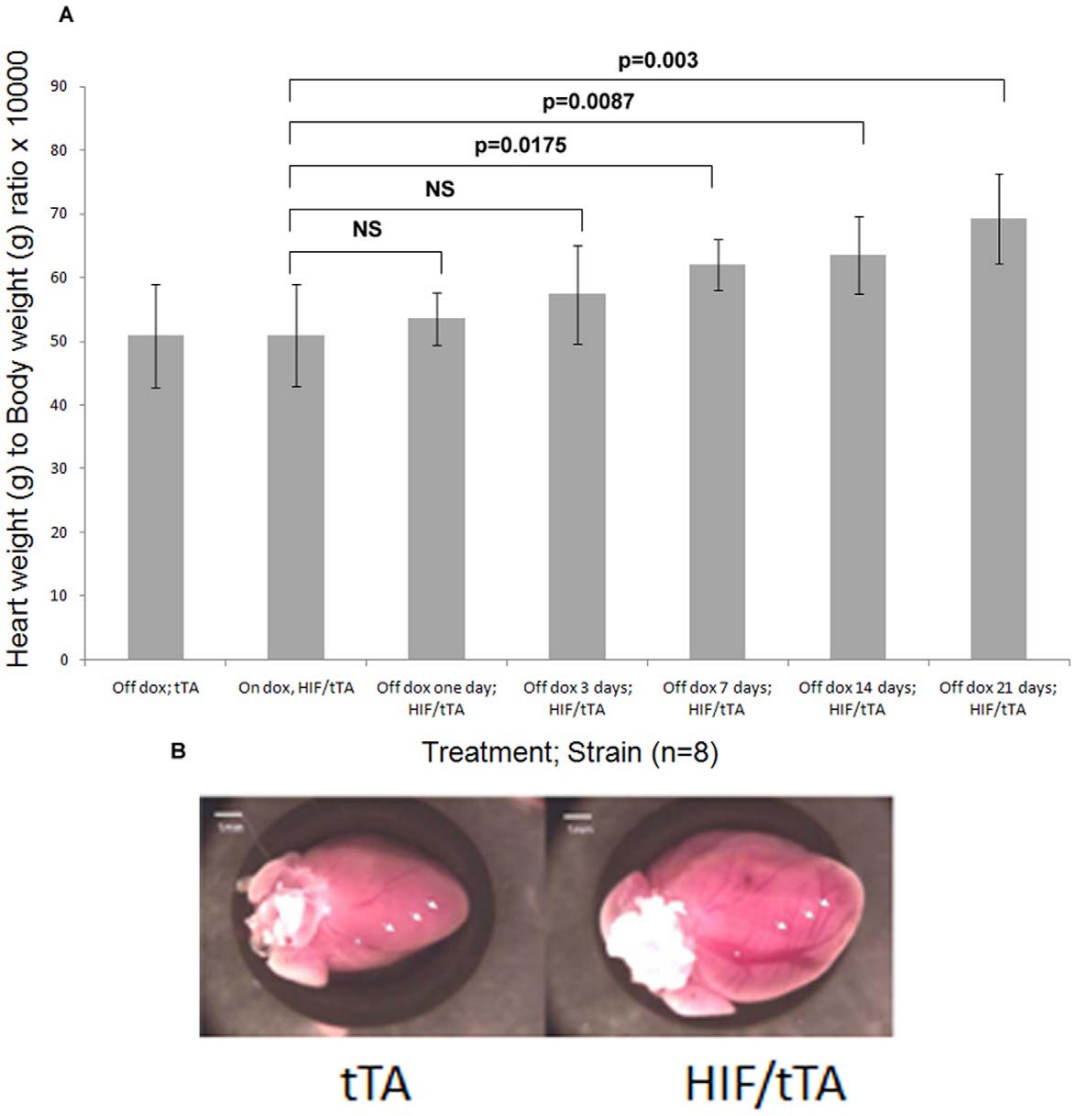
Changes in heart weight and epicardial vessels after HIF expression. (**A**) Heart weight to body weight ratio following doxycycline removal. (**B**) Macroscopic view of tTA heart compared to HIF/tTA seven days after doxycycline removal. Arrows point to epicardial blood vessels that are much more prominent after.

**Figure 5:**
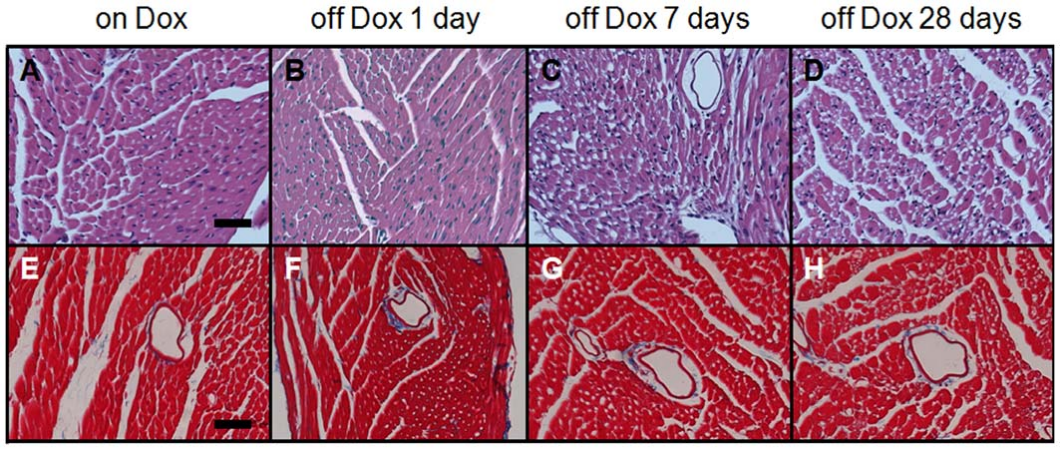
Representative histology of HIF-1α-PPN expressing hearts. Top panels: hematoxylin and eosin; Lower panels: trichrome, Scale bar: 50 µm.

Following removal of doxycycline from the drinking water, heart weight to body weight ratio size increased significantly (Figure 4). On initial macroscopic inspection, remarkably large epicardial vessels with prominent side branches were observed (Figure 4). The myocardium showed no obvious abnormality, specifically no heterogeneity in myocyte size, hypertrophy, or scarring, that might suggest myocyte death contributing to the ventricular dysfunction. Additionally, histological evaluation of the heart by hematoxylin and eosin or trichrome staining demonstrated no obvious pathology due to HIF-1α-PPN over-expression (Figure 5). TUNEL staining also showed no appreciable differences over twenty-eight days of HIF-1α-PPN expression (data not shown).

### Reversibility Study

**Figure 6:**
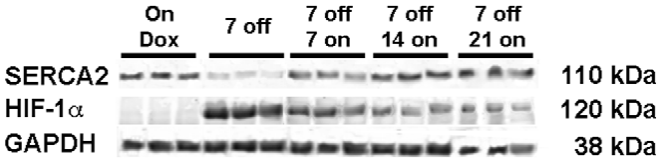
Western blot of HIF-1α-PPN and SERCA levels following doxycycline restoration.

After 7 days of removal of doxycycline followed by restoration of inhibition of the tet-transactivator protein for 7, 14 and 21 days, the heart weight to body weight ratio decreased gradually (7 day 0.0070±0.0016; 14 day 0.0052±0.0008 and 21 day 0.0055±0.0013) towards the ratio seen in the on-doxycycline control (0.0047±0.0002). Re-inhibition of HIF-1α-PPN expression was associated with an increase in SERCA protein in the myocardium (Figure 6). Full restoration to baseline levels was observed 14 days after doxycycline was restored, and this recovery was maintained through 21 days. The return of SERCA was temporally correlated with a restoration of cardiac function assessed by echocardiogram (see above). HIF protein levels decreased gradually after doxycycline was restored (to 41%±4% of baseline after 21 days), decreasing at a rate slower than SERCA restoration (Figure 6).

#### Cardiac gene regulation after expression of HIF-1α-PPN

**Figure 7:**
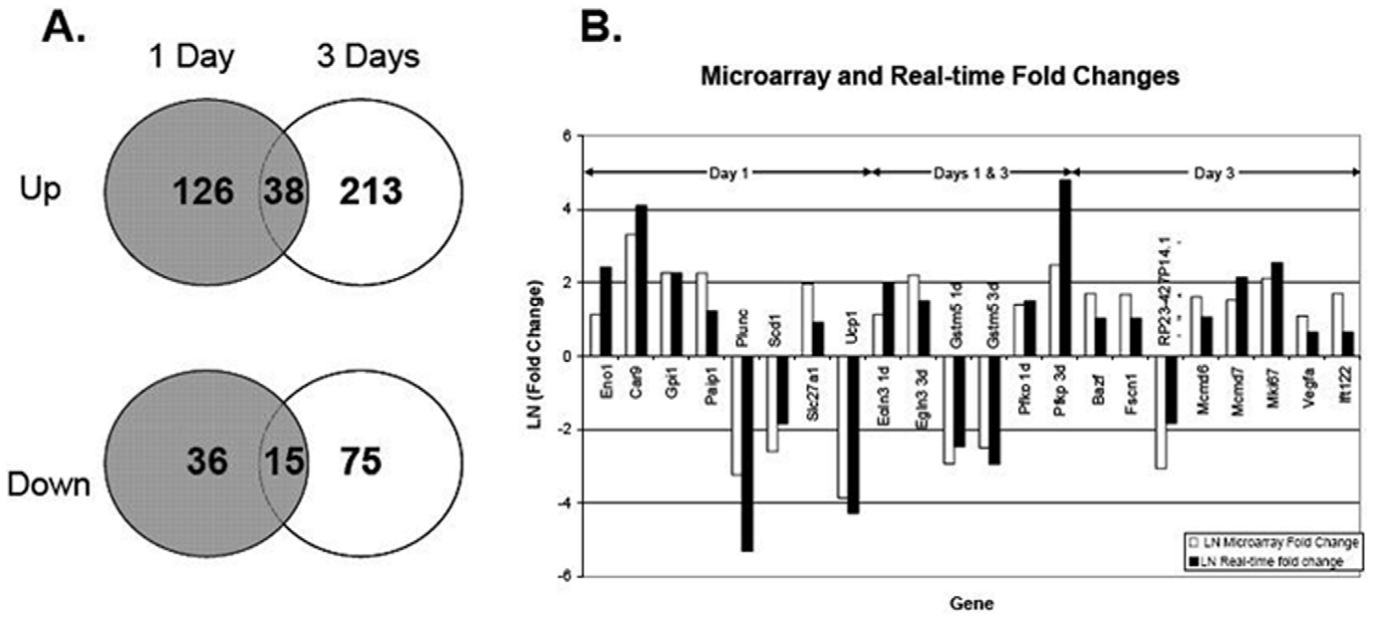
Transcriptional regulation by cardiac HIF. (**A**) Venn diagram of genes regulated >3-fold in the heart as determined by microarray. (**B**) RT-PCR Gene expression results for tTA/HIF-1α-PPN mice compared to tTA control mice.

Analysis of gene regulation by microarray analysis revealed a large number of genes that were both up- and down-regulated (>3-fold) after HIF-1α-PPN expression for either 1 day or 3 days (GEO reference GPL7119). The abundance of the message for 126 genes was more than 3-fold increased after 1 day of HIF expression in the heart. 213 genes were up-regulated at 3 days and 38 genes were common to both time-points (Figure 7A). Thirty-seven genes were down-regulated more than 3-fold at day 1 and 100 at day 3, with 3 common to both (Figure 7A). Inspection of the twenty most highly up-regulated genes at day 1 or day 3 of transgene expression showed no significant difference in the frequency of evolutionarily conserved HRE core sites (data not shown, p = 0.75). The expression levels of 19 notable and highly regulated transcripts from the array analysis were further confirmed by real-time PCR. Results from the real-time PCR experiments are shown in Figure 7B. As expected, the message for VEGF-A is approximately 2-fold more abundant. The other regulated genes shown in Figure 7B can be characterized as functioning in metabolism (Car9; Gstm5; Scd1; Slc27a1, Ucp1, Eno1; Gpi1; Pfkp), prolyl hydroxylation (Egln3), replication/transcription (Bazf; Mki67; Mcmd6; Mcmd7; Paip1), and other miscellaneous, or uncharacterized functions (Fscn1; RP23-427P14.1, Plunc, Ift122). In summary, we find that the abundance of mRNA from a large number of genes vital to glycolysis are increased, whereas the majority of mRNAs for genes necessary for oxidative phosphorylation are less abundant.

**Figure 8:**
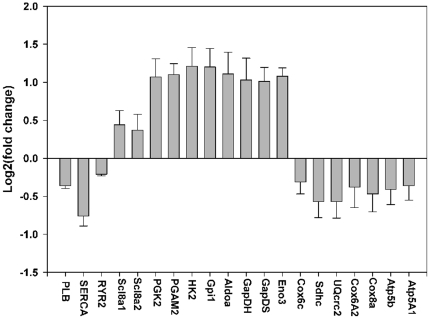
Semi-quantitative real-time PCR analysis of transcript abundance in hearts after HIF induction. Metabolism-related genes showing the expected up-regulation of glycolytic genes and down-regulation of genes involved in oxidative metabolism. Evaluation of select genes involved in calcium cycling shows down-regulation of SERCA, along with moderate down-regulation of RYR-2 and PLB.

Somewhat unexpectedly, an additional expanded evaluation focused on the transcription of genes important for calcium handling in the cardiomyocytes, showed that the mRNA for the sarcoplasmic endoplasmic reticulum calcium ATPase (SERCA) pump is half as abundant after 72 hours of induction of HIF-PPN (Figure 8). We also saw reduced abundance of the mRNA for phospholamban (PLB) and the type-2 ryanodine receptor (RYR2) (Figure 8).

### Chromatin immunoprecipitation

Chromatin immunoprecipitation identified direct binding of HIF-1α to six of the nineteen genes found to be dysregulated by real-time PCR and microarray analyses (Supplemental Table S1). An additional thirty genes, directly regulated by HIF-1α-PPN, have also been confirmed by both ChIP and microarray analyses (Supplemental Table S1).

### ATP:ADP ratio

**Figure 9:**
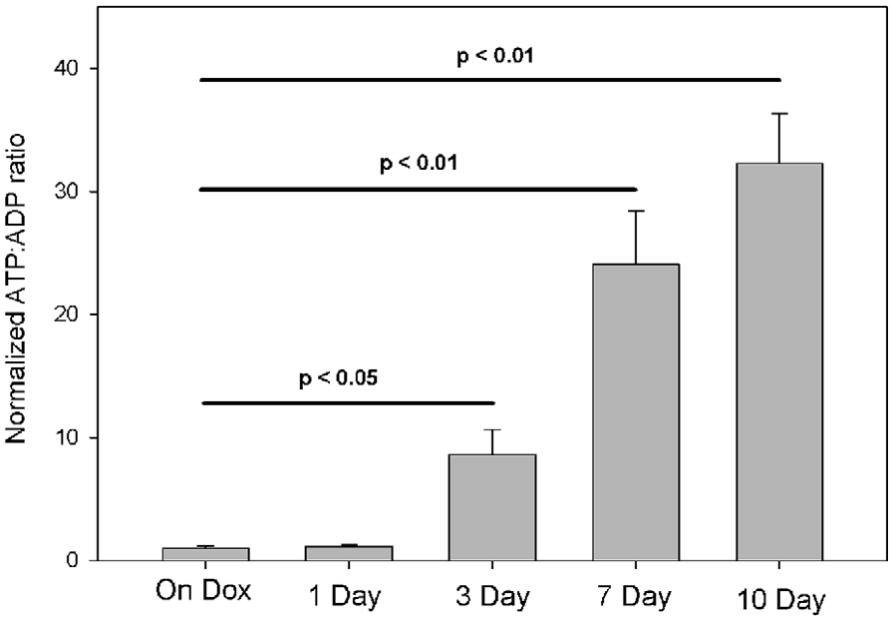
Effect of HIF expression on cardiac ATP:ADP ratio. Progressive increase in ATP:ADP ratio was observed after doxycycline was withheld in the hearts of HIF/tTA mice. (n =  three hearts per time point).

The transcriptional evaluation suggests an important (and expected) shift in metabolism from oxidative to glycolytic metabolism, permitting continued function with reduced oxygen consumption. Based on this consideration, we evaluated whether the ventricular dysfunction we saw could be the result of a defect in metabolism due to an inappropriate shift from oxidative phosphorylation to less efficient glycolysis. Surprisingly, despite the shift to potentially less efficient ATP production, the ATP:ADP ratio increased after HIF induction (Figure 9). This is primarily due to an increase in the available ATP within the myocardium, coupled with a relatively unchanged ADP concentration. We then considered the possibility that the cardiac dysfunction was not caused by reduced ATP availability, but conversely, that the changes we observed in ATP concentration resulted from reduced ATP utilization due to reduced contractility. Specifically, we considered the possibility that the HIF response, rather than reducing oxygen demand in the myocardium, was conserving ATP as a temporary measure to maintain viability during transient hypoxic episodes. We evaluated key players in excitation-contraction coupling in the heart to determine whether HIF-directed modification of this pathway could explain the loss of contractility.

### Direct measure of calcium flux

**Figure 10:**
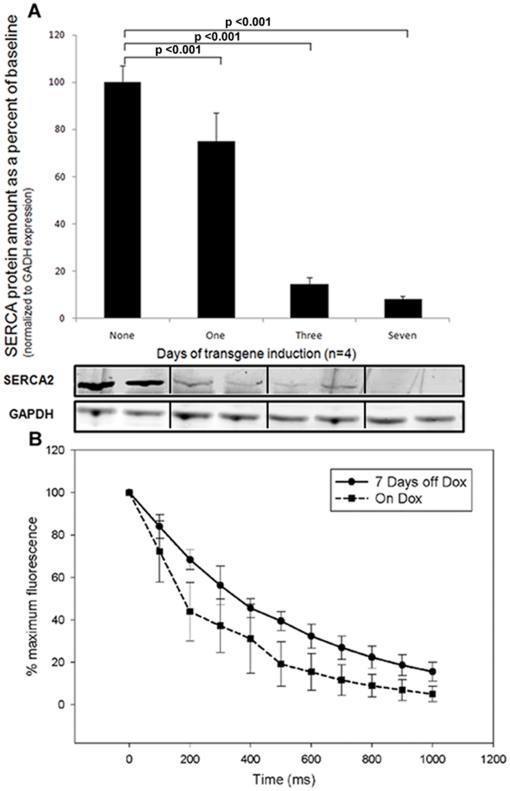
SERCA protein levels and calcium reuptake after HIF expression. (**A**) SERCA western blot. SERCA is markedly reduced after even a single day of transgene induction; the western blot below the graph shows results from two animals at each timepoint. (**B**) Calcium reuptake in cardiomyocytes is slower after transgene induction.

Our transcriptional evaluation did indeed demonstrate substantial down-regulation of the mRNA for SERCA. The abundance of cardiac SERCA mRNA was reduced 2-fold after 3 days of transgene expression and SERCA protein was reduced to an even greater degree (Figure 10). If re-uptake of calcium is depressed by reduced SERCA amount or activity, then reduced inotropy would be expected. We did find that Ca^++^ uptake was impaired after 7 days of oxygen-stable HIF expression and therefore ascribe at least a portion of the ventricular dysfunction to this cause. To quantify this slowed reuptake, the decay in calcium fluorescence was fit to an exponential decay model (see Method's section for further detail), and a quantity, τ, representing the time constant of decay, could thus be calculated. A higher value of τ is representative of slower calcium uptake. We found that τ increased from 312 +/− 39 ms to 526 +/− 35 ms, indicating the expected impairment of reuptake of Ca^++^ by SERCA.

## Discussion

We have created a transgenic model that allows the regulated expression of constitutively active HIF-1α in the adult myocardium. These animals allow us to identify the genes that are regulated by HIF in vivo, as well as the resulting physiologic, metabolic, and anatomic effects of HIF expression.

During HIF-1 expression in adult transgenic mice, ventricular function deteriorated and the heart dilated. Importantly, ventricular function returned to normal 7 days after transgene expression was terminated, indicating that the decrease in ventricular function was reversible and not related to irrevocable myocyte death. A recent study utilizing a cardiac-specific VHL KO −/− mouse also demonstrated a decline in ventricular function, manifesting at three months of age, that was prevented by an additional deletion of HIF-1α [24]. Taken together, our findings and these support the direct role of HIF-1α transcriptional activity in ventricular dysfunction, although it is certainly possible that the other study reflects activities of HIF during development or growth, that impair contractility by a distinct mechanism.

Our model allows us to examine the earliest gene regulation induced by HIF in vivo. These early genes are likely to be directly regulated by HIF, and thus provide insight into the pathways that transduce HIF effects. As seen in previous in vitro studies [29], [30], the mRNAs for several glycolytic enzymes, particularly Eno1 and Pfk, were up-regulated. HIF-1α-PPN expression also induced indicators of cellular proliferation, including Mki67 and Mcmd6 mRNAs. These transcripts may be related to the observed angiogenic response, occurring in the endothelial cells and pericytes recruited to new vessels. Several cancer-related transcripts were found to be up-regulated as well, including the mRNA for actin-bundling protein Fscn-1 (reviewed in [31]). Bcl-6, a homolog of the up-regulated Bazf gene, has been found to be up-regulated in breast cancer and associated with higher HIF-1α levels [32]. Many of the HIF-regulated genes in this study have not been previously recognized to be related to hypoxia, and their putative roles in the hypoxic response will require more evaluation.

The HIF-induced changes in mRNA abundance that we observe are consistent with the anticipated shift from oxidative phosphorylation to glycolytic metabolism. Importantly, we also see a reduction in the abundance of the mRNAs for key components of excitation-contraction coupling. In particular, we have observed reduced abundance of the mRNA for the SERCA2a calcium pump, as well as transcripts for PLB and RYR2. Down-regulation of these three genes would be predicted to produce a reduction in contractility in the heart due to a reduced calcium gradient across the SR membrane, and a reduced release of calcium upon initiation of contraction through the RYR2 channel. We propose that the heart cannot maintain normal contractility because of reduced expression of the SERCA pump, with resulting slowed re-uptake of cytoplasmic Ca^++^. Our direct measurement of calcium flux supports this mechanism.

In human hearts there is strong evidence for down-regulation of SERCA in ischemia[33]and heart failure[34]. There is an ongoing effort to develop methods to treat heart failure by modulating SERCA and other components of Ca regulation[35]. Our data further emphasizes the importance of reduced SERCA as a primary effector of cardiac contractility, and therefore a worthy target for such strategies. It is interesting in this regard that the gene for SERCA has several potential hypoxia response elements in its promoter and that the response to the single stimulus of HIF induction is rapid. Both observations suggest that HIF-may be directly regulating SERCA, although this promoter was not identified among the ChIP positive fragments.

The paradoxical change in the ATP:ADP ratio suggests that the upregulated glycolytic pathway is able to maintain ATP stores. Reduced calcium cycling from the sarcoplasmic reticulum, which we expect with reduced SERCA, could be responsible for both reducing contractility and maintaining the ATP:ADP ratio by reducing ATP consumption during the cardiac cycle.

In patients with ischemic cardiomyopathy, PET studies have demonstrated that hibernating myocardium, defined as areas of the heart with reduced blood flow but maintained glucose utilization, is less common than “stunned” myocardium with preserved blood flow. Nonetheless, 9% of dysfunctional segments in such patients show the reduced perfusion, (with preserved glucose metabolism), that would be expected to lead to chronic HIF stabilization [36]. Parenthetically, it may be that the shift to glycolysis expected with HIF action is playing a role in preserving glucose utilization in these segments of underperfused heart. If HIF is stabilized in these segments it could be responsible for the impaired contractility, as seen in our mouse model [28].

Our findings suggest that a portion of the reduced contractility in ischemic cardiomyopathy could be directed by HIF. Our model allows us to study HIF in isolation from other transcription factors that may be active during an ischemic insult. The data presented here suggest that HIF, acting alone upon the heart, causes cardiac dysfunction. The mechanism is at least in part mediated by reduction in calcium reuptake into the sarcoplasmic reticulum.

A limitation of our approach is that we have examined the action of HIF in normoxic tissue, whereas the authentic in vivo action of HIF will occur predominately in hypoxic tissue where redox potential, pH, and substrate utilization may modify the transcriptional activity of HIF. However, the *in vivo* effects that we see are almost certainly more authentic than those obtained in cell culture, and seem likely to accurately recapitulate effects of human cardiac ischemia that occurs in mature adults.

The principal finding of this study, the unexpected, reversible, decrement in ventricular function, must be considered in any effort at HIF-1α-directed therapy. Perhaps of even greater clinical significance, this finding suggests a novel mechanism for ischemic cardiomyopathy. If persistent ischemia produces chronic HIF stabilization in even a portion of the ischemic heart, then the action of this transcription factor, perhaps acting in trans through downstream intra-cardiac signaling pathways, could directly impair ventricular performance. An exciting ramification of this hypothesis is that new therapy directed at HIF action could ameliorate this dysfunction.

## Methods

### Transgenic animal generation

#### Ethics Statement

All animals were handled in accordance with good animal practice as defined by the relevant national and/or local animal welfare bodies, and all animal work was approved by the appropriate committee (University of Hawaii Institutional Animal Care and Use Committee, approval number 06-011-4). PCR-based mutagenesis was used to create a mutated human HIF-1α transgene (residues Pro402, Pro564, and Asn803 to Ala, HIF-1α-PPN) that was cloned into the pcDNA 3.1 vector (Invitrogen, Carlsbad, CA). A hemagglutinin (HA) tag was added after the last c-terminal codon using a linker (between Hpa I and Xba I). This plasmid was used for transfection of tissue culture cells, as well as for production of an adenoviral vector encoding HIF-1α-PPN under the regulation of the cytomegalovirus (CMV) promoter. The HA-tagged HIF-1α-PPN cDNA was subcloned into the tetracycline-responsive pUHG-10 vector to generate the pUHG-HIF-1α-PPN vector. DNA was injected into B6C3F1 mouse oocytes and implanted into surrogate female ICR mice. Founder mice were identified by PCR with a reverse primer located in the HA-tag. Transgenic offspring were bred to a second transgenic line containing the tetracycline trans-activator region fused downstream of the cardiac-specific α-myosin heavy chain (αMHC) promoter [the generous gift of Dr. Glen Fishman]. Double transgenic mice (tTA/HIF-1α-PPN) were maintained on 200 µg doxycycline per ml of 2.5% sucrose-water to suppress HIF-1α-PPN expression. All animals were treated with doxycycline from conception to 6 weeks. Thereafter, doxycycline was omitted for varying periods to assess experimental effects of the mutant HIF-1α transgene. All experiments used 6 to 8 week old male mice.

### HIF-inducible luciferase assay

Constitutive normoxic induction of the HIF-1α-PPN mutant was assessed by transient transfection of HeLa cells with increasing amounts of either a wild-type HIF-1α expression vector or the PPN expression vector. HIF activity was measured by induction of a HIF-inducible luciferase reporter driven by three tandem HIF response elements [3HRE-tk-luc[37]]. Activity of this promoter following hypoxic induction of endogenous HIF (15 h at 1% O_2_) was used to assess maximal endogenous regulation. Luciferase activity was measured using Luciferase Reporter Assay System (Promega, Madison, WI), following manufacturer's instructions, 20 hrs following transient transfection using the Lipofectamine PLUS reagent (Invitrogen, Carlsbad, CA).

### *In vitro* experiments using an adenoviral vector

An adenoviral vector encoding the HA-tagged cDNA of HIF-1α-PPN under the control of a CMV promoter was generated (AdCMV-HIF-1α-PPN). The virus was used to transfect cultured mouse endothelial cells (SVEC) and rat neonatal cardiac myocytes to evaluate expression of the protein by Western blotting. Plates with 1.3×10^6^ cells were incubated with 200 pfu/cell of AdCMV-HIF-1α-PPN or the same amount of AdCMV-pLpA (no cDNA) as a control. Cells were harvested after 72 h and total protein lysates were prepared. Western blots were performed after isolation and concentration of the protein by magnetic immunoprecipitation. The abundance of the VEGFa mRNA was determined by real-time PCR.

### Chromatin Immunoprecipitation Assays (ChIP)

After one or three day induction of the transgene, DNA from individual mouse hearts (n = 3 for each time point) was subjected to magnetic-ChIP (Millipore, Billerica, MA), according to manufacturers protocol, using an antibody to the HA tag. Fragments immunoprecipitated from the ChIP assay were ligated into pZEr0-1 plasmids, cloned into TOP 10 *E.coli* (all Invitrogen, Carlsbad, CA), and sequenced using plasmid-specific T7 sequencing primers. Resulting sequence was mapped to the mouse genome (UCSC genome browser, July 2007), following nucleotide BLAST analyses (NCBI).

### Immunoprecipitation and Western Blotting

Individual whole mouse hearts were homogenized in RIPA-buffer and incubated for 1 h at 4°C. Following centrifugation, 500 µl of the supernatant was cleared by magnetic immunoprecipitation using a commercial kit with anti-HA-microbeads according to the manufacturer's protocol (Miltenyi Biotec, Auburn, CA). Western blots were performed with either a primary monoclonal anti-HA antibody (100 ng/ml) (Roche, Mannheim, Germany), a monoclonal anti-human-HIF-1α antibody (1∶500) (Novus Biologicals, Littleton, CO), or a monoclonal goat anti-SERCA2 antibody (1∶1,000) (Santa Cruz, Santa Cruz, CA). After incubation with a secondary peroxidase-conjugated antibody (for HA and HIF-1 α), or AlexFluor®-568-conjugated antibody (for SERCA2) (Invitrogen, Carlsbad, CA), blots were developed using a commercial substrate (SuperSignal West Pico, Pierce, Rockford, IL) and visualized with a Typhoon scanner (GE Lifesciences, Piscataway, NJ) (535 nm excitation, 560LP emission, 500PMT). ImageJ software was used for densitometry analyses.

### Echocardiography

We evaluated left ventricular function in unsedated mice with transthoracic echocardiography (Sonos 5500 machine, Philips, Andover, MA) using a S12 transducer (12 MHz). We examined both tTA/HIF-1α-PPN and tTA mice at various times after omitting or adding back doxycycline (n = 4 for each strain at each time point). Left ventricular parasternal short-axis views were obtained in M-mode imaging at the level of the papillary muscle. Two consecutive beats in three M-mode images (6 beats total) were used for measurements of left ventricular end-diastolic internal diameter (LVEDD), left ventricular end-systolic internal diameter (LVESD), and posterior wall thickness (PWT). Fractional shortening (FS) was calculated as FS% = [(LVEDD-LVESD)/LVEDD]×100.

### Reversibility study

Mice were taken off doxycycline for 7 days, at which time three hearts were harvested to assess activation of HIF-1α-PPN, and doxycycline was returned to the drinking water of all remaining mice. Three hearts were obtained at 7, 14 and 21 days following restoration of doxycycline. At the end of the experiment, control hearts from mice that had been maintained on doxycycline were obtained. Whole heart lysates were prepared as described above, and Western blot analysis was performed to determine protein abundance of HIF-1α-PPN and SERCA2. Protein abundance was quantified using densiometry and was normalized to GAPDH abundance.

### Immunohistochemistry and histology

Mice (n = 3 for each evaluation) were deeply anesthetized with Ketamine/Xylazine, then perfused with PBS by cardiac puncture followed by fixation with 15 mL of 4% paraformaldehyde in PBS. For paraffin-embedded tissue, hearts were removed and immersed in 4% paraformaldehyde for 4 hours, then transferred to 70% ethanol. Following dehydration through ethanol and transfer to xylene, hearts were embedded in hot paraffin wax. Five mm sections were cut with a microtome and kept on a heat block overnight. Prior to staining, slides were deparaffinized in xylene and rehydrated in an ethanol series.

For hematoxylin & eosin staining, slides were incubated in Gill number 2 hematoxylin (Richard Allen Scientific) for 3 minutes, rinsed under running tap water, then counterstained with eosin Y (EMD) for one minute, and finally rinsed for one minute in acetic water (0.1% glacial acetic acid in water). Masson's trichrome staining was performed using The Trichrome Modified Masson's Stain Kit (Scytek Labs), according to manufacturer's instructions. Following trichrome and H&E staining, slides were dehydrated in an ethanol series, cleared in xylenes, and coverslipped with Permount (Fisher) mounting medium.

### RNA preparation, microarray analysis, and real-time PCR

Total RNA from mouse hearts was isolated with TRIzol reagent (Invitrogen, Carlsbad, CA) according to the manufacturer's protocol. For microarray analysis, probes were generated from reverse transcription of 20 µg of pooled RNA for each of tTA and tTA/HIF-1α-PPN sample groups (n = 3 per time point) with Cy3 and Cy5. Hybridization was performed overnight against arrays containing the 32,000 murine long-oligo V3 collection (Qiagen, Valenica, CA). A Genepix system (Axon Instruments, Union City, CA) and Gene Traffic software (Iobion, La Jolla, CA) were used for scanning and analyzing data, respectively.

For real-time PCR, cDNA was produced from each individual heart (n = 3 for each time point) to allow assessment of inter-animal variation. Oligonucleotide primers were designed to cross an intron. Real-time PCR was performed on cDNA representing 5 ng of total RNA. PCR was run in triplicate with QuantiTect SYBR Green (Qiagen, Hilden, Germany) in an Opticon device (MJ Research, Waltham, MA). Tangerin, which was unregulated on initial array analyses, was used as an internal control.

### HRE Scanning

Genes directly regulated by HIF-1 are likely to contain a core HRE site 5′-RCGTG-3′ [30]. In order to evaluate whether or not the genes regulated shortly after HIF expression were more likely to contain such a canonical HRE, we examined the 20 most up-regulated genes identified after 1 or 3 days of HIF expression. In order to enhance the specificity of our survey, we focused on core HRE binding sites that were conserved between the mouse and human genomes. The rVista 2.0 program [38] was used to align and scan each gene for potential conserved HRE core sites located within non-coding regions between 2 kb upstream and 1 kb downstream of the gene. We counted sites located within evolutionarily conserved regions at least 100 base pairs in length having at least 70% inter-species conservation. Additionally, local conservation, evaluated as at least 80% identity in a 20 base-pair sliding window containing the core site, was required of the potential HRE sites. Significance was tested with Fisher's exact test.

### ATP:ADP ratio

Whole heart cell lysates (n = 3) were analyzed using a Bioassay Systems Enzylight™ ATP:ADP ratio kit (ELDT-100, Bioassay Systems, Hayward, California). 5 µL of cell lysates, normalized to a protein concentration of 5.0 mg/mL by Bradford analysis, were treated with two compounds which generated light in direct proportion initially to the concentration of ATP, and subsequently to the sum of the ATP and ADP signal. The ratio between the two compounds is calculated from the difference between the two measurements, divided by the ATP signal. Luminometry was assessed using a TD-20/20 Luminometer (Turner Designs, Sunnyvale, California). RLU counts were integrated over 5 seconds, after a 2 second wait time.

### Calcium Flux

After anesthesia, hearts (n = 3) were excised from adult male mice and flushed with a Tyrodes' solution containing 100 µM Ca^2+^ through which 95% oxygen had been bubbled. The ventricles were minced and digested with 300 µg/mL of Liberase Blendzyme 2 (Roche) for 1 hour at 37 C with trituration every 5 minutes. Cells were then transferred to DMEM containing 10% fetal calf serum to halt the enzymatic reaction. The cells were plated onto laminin-coated glass-bottomed culture dishes (MatTEK Corp., Ashland, MA). After 24 hours, the media was replaced with DMEM solution containing 3.0 µM of Fluo-4 calcium indicator dye (Invitrogen, Carlsbad, CA). After one hour of incubation, the cells were stimulated with 100 nM isoproterenol, and fluorescence intensity was measured along a line perpendicular to the long axis of the cell at a frequency of 10 hz using a Zeiss LSM-5 Pascal Laser Scanning Microscope (Zeiss, Jena Germany). Fluorescence was measured on an arbitrary scale where the background fluorescence was zero, and the maximum measured fluorescence was equivalent to 1. A single-variable least-squares algorithm was created to fit the fluorescence values to an equation of the form: f = f_o_e^t/τ^. Where f is the fluorescence at any given time t, and f_0_ is the maximum fluorescence elicited during a cardiac cycle. The activity of the SERCA pump is the primary pathway by which calcium is removed from the cytoplasm. Therefore, a higher calculated value of τ corresponds primarily to a reduced cellular activity of SERCA.

## Supporting Information

Table S1 Select genes from microarray analyses confirmed by ChIP assays.
(0.06 MB DOC)
