# Supplementary material for: Conditional HIF-1α Expression Produces a Reversible Cardiomyopathy
Source: PLoS One. 2010 Jul 21;5(7):e11693. doi: 10.1371/journal.pone.0011693 (PMC2908132; doi:10.1371/journal.pone.0011693)
Supplement: Table S1 — Select genes from microarray analyses confirmed by ChIP assays. (0.06 MB DOC) [file pone.0011693.s001.doc]

**Table S1. Select genes from microarray analyses confirmed by ChIP assays**

| | **Gene** | **Days of induction** | **rVISTA confirmed HRE location** | | --- | --- | --- | | Eno1^*^ | One | 3’ UTR | | Car9^*^ |  |  | | Gpi1* |  | 5’ UTR | | Paip1^*^ |  |  | | Plunc* |  |  | | Scd1* |  | 3’ UTR | | Slc27a1* |  |  | | Ucp1* |  |  | | Wisp1 |  | 5’ UTR | | Vwf |  | 5’ UTR | | Usp3 |  | 5’ UTR | | Pdgfra |  | 5’ UTR | | Eef1g |  | 5’ UTR | | Rgs3 |  | 5’ UTR | | Prkcbp1 |  | 5’ UTR | | Ncoa3 |  |  | | NM_145484 |  |  | | Mapre2 |  |  | | Fbxl18 |  |  | | Ada |  |  | | Slc30a10 |  | 5’ UTR | | Zfp469 |  | 5’ UTR | | LOC208820 |  |  | | LOC68099 |  |  | | LOC76862 |  |  | | LOC269695 |  | 5’ UTR | | LOC545279 |  | 5’ UTR | | LOC211208 |  |  | | Egln3* | One and three | 5’ UTR | | Gstm5* |  | 3’ UTR | | Pfkp^*^ |  | 5’ UTR | | Abcg1 |  | 5’ UTR | | Pcca |  | 5’ UTR | | Car12 |  | 5’ UTR | | A1bg |  | 5’ UTR | | E2G 2 |  | 5’ UTR | | Bazf* | Three |  | | Fscn1^*^ |  |  | | Mcmd6* |  | 5’ UTR | | Mki67* |  |  | | Vegfa^*^ |  | 5’ UTR | | Mcmd7* |  |  | | Ift122* |  |  | | RP23-427P14.1* |  |  | | Eif3s10 |  | 5’ UTR | | L36a |  | 5’ UTR | | NM_146106 |  |  | |  |  |
| --- | --- | --- | --- | --- | --- | --- | --- | --- | --- | --- | --- | --- | --- | --- | --- | --- | --- | --- | --- | --- | --- | --- | --- | --- | --- | --- | --- | --- | --- | --- | --- | --- | --- | --- | --- | --- | --- | --- | --- | --- | --- | --- | --- | --- | --- | --- | --- | --- | --- | --- | --- | --- | --- | --- | --- | --- | --- | --- | --- | --- | --- | --- | --- | --- | --- | --- | --- | --- | --- | --- | --- | --- | --- | --- | --- | --- | --- | --- | --- | --- | --- | --- | --- | --- | --- | --- | --- | --- | --- | --- | --- | --- | --- | --- | --- | --- | --- | --- | --- | --- | --- | --- | --- | --- | --- | --- | --- | --- | --- | --- | --- | --- | --- | --- | --- | --- | --- | --- | --- | --- | --- | --- | --- | --- | --- | --- | --- | --- | --- | --- | --- | --- | --- | --- | --- | --- | --- | --- | --- | --- | --- | --- | --- | --- | --- | --- |

^*^Confirmed by real-time PCR

UTR; untranslated region
